# Impact of epilepsy surgery on the adaptive behavior of children with drug‐resistant epilepsy

**DOI:** 10.1111/epi.18437

**Published:** 2025-04-29

**Authors:** Ana Valeria Duarte Oliveira, Hélio Rubens Machado, Úrsula Thomé, Marcelo Volpon Santos, Geisa de Angelis, João Pereira Leite, Antonio Carlos dos Santos, Lauro Wichert‐Ana, Ana Paula Hamad, Américo Ceiki Sakamoto, Tonicarlo Rodrigues Velasco

**Affiliations:** ^1^ Center for Pediatric Epilepsy Surgery, University Hospital, Ribeirão Preto Medical School University of São Paulo Ribeirão Preto Brazil; ^2^ Division of Pediatric Neurosurgery, Ribeirão Prêto Medical School University of São Paulo Ribeirão Preto Brazil

**Keywords:** pediatric surgery, seizure control, Vineland scale

## Abstract

**Objective:**

This study was undertaken to assess the impact of surgical treatment on the adaptive abilities of children with drug‐resistant epilepsy (DRE) and moderate or severe developmental delays, and to identify factors that could potentially predict adaptive outcomes following epilepsy surgery.

**Methods:**

A retrospective observational cohort study was conducted involving 130 pediatric patients with DRE treated in the Ribeirão Preto Epilepsy Surgery Program of the University of São Paulo between 1996 and 2019. Patients underwent comprehensive preoperative evaluations, including neurological, psychiatric, social, and neuropsychological assessments using the Vineland Adaptive Behavior Scale. Adaptive functioning was assessed at three time points: before surgery and approximately 15 months and 34 months after surgery. Seizure outcomes were classified using the Engel scale. Statistical analyses included analysis of variance, Spearman correlation, and general linear model for repeated measures.

**Results:**

Before surgery, patients exhibited severe adaptive delays, with an average age equivalence (AE) of 18.2 months compared to an average chronological age of 78 months. Postoperatively, significant improvements in AE were observed, particularly among patients who achieved seizure freedom (Engel class I). At the first postoperative evaluation (median of 15 months after surgery), the average AE increased to 24.1 months (*p* < .01). At the second postoperative evaluation (median of 34 months after surgery), the average AE further increased to 27.5 months. Seizure‐free patients demonstrated greater improvements in adaptive skills compared to those who continued to experience seizures (*F* = 5.845, *p* = .018)

**Significance:**

This study reinforces that epilepsy surgery can lead to significant adaptive improvements in children with DRE, including those with severe neurological impairments. The findings highlight the positive impact of seizure freedom on developmental progress and underscore the importance of early intervention to minimize adaptive delays.

**Key points**

- This study highlights the significant delay in adaptive development, assessed as moderate or severe, determining the impact of epilepsy surgery on this population.
- Epilepsy surgery is promising in terms of adaptive functioning for pediatric patients with drug‐resistant epilepsy, including those with severe neurological impairments.
- The difference in scores between chronological age and age equivalence highlights the importance of early surgical intervention to maximize developmental outcomes.
- Seizure control as assessed by the Engel scale is a key factor in improving adaptive outcomes following epilepsy surgery.

## 1 INTRODUCTION

Epilepsy is a significant and common neurological disorder that can affect children during their developmental stages, particularly within the first year of life.^1^ The occurrence of epilepsy in early childhood raises concerns about potential impairments in brain development.^1^, ^2^ This age group is particularly susceptible to severe forms of epilepsy, including developmental and epileptic encephalopathies.^1^ Approximately 10% of epilepsy cases begin within the first 3 years of life,^3^ and overall, epilepsy affects at least 1% of individuals before they reach the age of 20 years.^4^

The early onset and frequent occurrence of seizures significantly increase the risk of children developing neuropsychomotor developmental dysfunctions and may lead to an unfavorable long‐term prognosis.^5^ Approximately 60%–70% of children with recent onset epilepsy can achieve seizure control using low to moderate doses of antiseizure medications (ASMs). However, a considerable proportion of patients—approximately 30%–40%—do not respond to treatment due to difficulties in controlling seizures, indicating the presence of drug‐resistant epilepsy (DRE).^6^ Another study reports that 20%–30% of children with epilepsy may experience DRE,^7^ and 10%–40% will continue to have DRE throughout their lives.^8^

The major impairments in these patients are cognitive, motor, language, and behavioral, which directly affect their daily functioning.^5^, ^9^ When DRE has an early onset and drug therapy does not satisfactorily control seizures, these patients have a higher risk of developing intellectual disorders and become candidates for epilepsy surgery.

Epilepsy surgery is supported by studies showing the benefits of surgical treatment compared to pharmacotherapy, including the potential for early successful intervention leading to remission of seizures. Early surgical intervention has proven to be effective, allowing 52%–80.3% of the pediatric population to become seizure‐free.^10^, ^11^, ^12^, ^13^, ^14^, ^15^, ^16^, ^17^ Developmental improvement is also related to the underlying pathology; children with acquired or progressive lesions have a more promising prognosis than those with cortical developmental malformations.^18^ However, there is a lack of evidence about the impact of epilepsy surgery in patients with major developmental impairments.

In a retrospective study, we aimed to assess the impact of surgical treatment on the adaptive abilities of children with DRE and moderate or severe developmental delays. Additionally, we aimed to identify factors that could potentially predict adaptive outcomes following epilepsy surgery.

## 2 MATERIALS AND METHODS

### 2.1 Patient selection

The study was classified as a retrospective observational cohort analysis. The study population consisted of 130 pediatric patients diagnosed with DRE, defined as a failure of adequate trials of two tolerated and appropriately chosen and used ASM schedules (whether as monotherapies or in combination) to achieve sustained seizure freedom.^19^ These patients were treated at a single center (Ribeirão Preto Epilepsy Surgery Center of University of São Paulo), by the same team, between 1996 and 2019.

Patients underwent presurgical evaluation to determine the location of the seizure. Exclusion criteria included patients whose parents or legal guardians had psychiatric disorders and those unable to complete the neuropsychological assessment questionnaire. At the start of the study, parents were interviewed, and medical records were reviewed. Prior written informed consent was obtained from the families before their inclusion in the study. All procedures were approved by institutional review boards.

Our inclusion criteria did not specifically exclude children with moderate delays; rather, the eligibility was based on the presence of developmental delay as defined by our assessment tools. Although we anticipated a broader range of severity (i.e., moderate, severe, and profound delays), the final study sample happened to have only children with severe or profound delays.

### 2.2 Preoperative evaluation

The preoperative evaluation was conducted by a properly trained multidisciplinary team, following a standardized protocol for specialist consultations and complementary examinations, such as video‐electroencephalographic (video‐EEG) monitoring and structural and functional neuroimaging, as published before.^20^, ^21^, ^22^, ^23^

During neurology consultations, parents or caregivers were interviewed to obtain clinical information regarding the patients, including seizure frequency, age at seizure onset, use of ASMs, family history, perinatal and developmental history, known genetic conditions, and comorbidities, such as psychiatric disorders and developmental delays.

All 130 patients included in the study underwent 1.5‐ or 3.0‐T magnetic resonance imaging (MRI). The etiology of epilepsy was based primarily on the presurgical MRI. However, we also assessed the histopathology report to confirm or refine the MRI diagnoses. Video‐EEG was performed to record ictal and interictal brain electrical activity using 10–20 or 10–10 electrode placement and reviewed in a Nihon‐Kohden System. Single‐photon emission computed tomography was utilized during ictal and interictal phases to localize the epileptogenic zones, using the radiopharmaceutical ethyl cysteinate dimer (ECD)‐technetium. Additionally, positron emission tomography was performed using fluorodeoxyglucose–glucose to assess brain metabolism.

The psychiatric assessment focused on identifying associated psychiatric comorbidities. The social assessment aimed to evaluate factors influencing the quality of life of both the patient and their family, including socioeconomic status, family routine, access to treatment, and school inclusion.

The neuropsychological assessment focused on analyzing cognitive functions, memory, language, and adaptive skills using standardized tests and developmental scales. In this study, patients eligible for epilepsy surgery exhibited a certain degree of cognitive impairment or, due to their chronological age, were not able to participate in a full battery of neuropsychological tests. Therefore, scales such as the Vineland Adaptive Behavior Scale (VABS) was employed to assess adaptive skills. The VABS consists of a semistructured interview with parents or caregivers, in which questions are asked regarding the patient's communication, socialization, daily living skills, and motor skills.^24^

### 2.3 Vineland Adaptive Behavior Scale

#### 2.3.1 VABS preoperative evaluation

Participants, ranging from 0 to 18 years of age, underwent neuropsychological assessments at three distinct time periods (presurgical, postsurgical I, and postsurgical II). During the evaluation, conducted at the Ribeirão Preto Epilepsy Surgery Program, it was observed that many children who were candidates for epilepsy surgery exhibited significantly delayed developmental levels or, due to their young age, lacked the necessary comprehension to fully participate in standard psychological assessments. In such cases, based on established protocols used by national and international epilepsy surgery centers, the VABS was administered as an alternative to assess adaptive development. This scale was deemed appropriate for evaluating the adaptive functioning of pediatric patients in these cases. The VABS used in this study is a semistructured caregiver interview consisting of 261 items that assess four key domains: communication, daily living skills, socialization, and motor skills. The scores were expressed as age equivalence (AE) scores, which represent the pediatric patients' developmental functioning relative to other pediatric patients of the same chronological age. We also analyzed the correlation of AE among the etiological groups during this phase.

To establish scoring, points from each domain were recorded in the evaluation form. The item scores reflect whether the patient performs the described activity, with a score of 2 indicating “yes, usually,” 1 indicating “sometimes or partially,” and 0 indicating “no, never.” The AE score was used at all research stages, where the total points in each domain were compared against the VABS manual, determining the chronological AE at the time of assessment.

All procedures related to VABS administration were conducted following the official translated manual that accompanies the scale. The authors who performed the statistical analysis did not participate in the administration of the VABS at any stage of this study; the assessments were exclusively conducted by the hospital's neuropsychologists. During the span of the study (between 1996 and 2019), more than one neuropsychologist administered the VABS. However, the same trained individual administered the VABS before and after surgery. Also, the neuropsychologist who administered the VABS was blind to other examinations, such as MRI results, type of surgery, and seizure outcome.

After the preoperative evaluation and confirmation of surgical eligibility, each case was reviewed and discussed during a multidisciplinary clinical meeting. The purpose of this meeting was to make a collective decision regarding whether to approve surgery, deny it, or recommend further invasive evaluations to localize the epileptogenic zone and determine surgical candidacy. In cases where surgery was deemed appropriate, the specific details of the procedure, as well as potential risks and benefits, were thoroughly determined.

Subsequently, a feedback meeting was held with the patients and their families, during which the team presented the decision to proceed with surgery. A clear explanation of the risks and benefits was provided, and families were encouraged to ask questions. If they agreed to proceed, they signed an informed consent form, acknowledging their understanding of and consent to the surgery.

### 2.4 Postoperative data (phases 1 and 2)

In the postoperative phase, we analyzed at two distinct time periods: postoperative evaluation I (median 15 months after surgery), and postoperative evaluation II (median 34 months after surgery). The VABS was specifically selected by the epilepsy surgery team at the Clinical Hospital of the Medical School of Ribeirão Preto–University of São Paulo (HCFMRP‐USP) to evaluate the patients' behavior after surgery and to measure potential improvements in communication, daily living skills, socialization, and motor skills following the surgical procedure.

Regarding the VABS assessment of motor skills, there is a specific consideration for this domain; it is conducted with pediatric patients from birth up to 5 years, 11 months, and 30 days, or optionally for individuals older than 6 years. In the standardization studies of the VABS edition used in our study, it was observed that there is minimal development beyond this age range, which is why the motor skills assessment is not performed once the age limit is exceeded. Therefore, for patients who did not undergo the motor skills assessment, only the averages of communication, socialization, and daily living skills were considered.

Of the initial group of 130 surgical patients, 63 did not undergo the postoperative motor skills assessment, resulting in a sample size of 67 patients. No new participants were recruited to replace those lost to follow‐up, and the analysis was conducted using the available data from the patients who attended the assessments.

### 2.5 Seizure outcomes

Seizure outcomes were documented based on caregiver reports and medical records. After the surgical procedure, patients returned to the HCFMRP‐USP Children's Epilepsy Infirmary for their first postoperative evaluation. Follow‐up visits were then scheduled at 2 months, 3 months, 6 months, and 1 year postsurgery to monitor seizure control and medication usage. These regular visits allowed for continuous assessment of patient progress, ensuring that necessary adjustments to their treatment plans could be made as needed.

Postoperative seizure outcomes were evaluated using the Engel classification scale.^25^ Each patient's Engel class was documented throughout their follow‐up, but only the most recent classification, as recorded in the medical records, was used for analysis and entered in the SPSS database.

Engel class I patients were those who remained seizure‐free following surgery. Engel class II were those who experienced occasional disabling seizures or were nearly seizure‐free. Patients who exhibited minor but worthwhile improvement in seizure control were classified as Engel class III. Finally, those with no change or seizure increase after surgery were classified as Engel class IV.

### 2.6 Statistical analysis

The normality of the variables was assessed using the Kolmogorov–Smirnov test and the chi‐squared test. Most of the VABS variables did not follow a normal distribution. Despite the large sample size (*N* = 130) and the nature of the data, we considered using nonparametric tests to ensure reliable results by meeting the necessary statistical requirements for accurate analysis.

To standardize the AE scores among the pediatric population, a statistical normalization process was applied. This allowed for the comparison of developmental delays or advances across patients of different chronological ages. The steps involved in normalizing the AE scores were as follows:

1. Deviation calculation: The AE scores and chronological ages of all participants were collected. For each patient, the deviation between the AE and chronological age was calculated as follows: deviation=AE–chronologicalage.
2. *Z*‐score calculation: The mean (*μ*) and SD (*σ*) of the deviations were computed, and each deviation was transformed into a *z*‐score to represent the number of SDs the value was above or below the mean:

$$Z=\frac{d\text{eviation}-\mu \;\left(\text{mean}\right)\;}{\sigma \;\left(\mathrm{SD}\right)}$$

*Z*‐scores close to 0 indicated that the AE was aligned with the group's average chronological age. Negative *z*‐scores suggested that the AE was below the average, indicating developmental delay. Positive *z*‐scores indicated that the AE was above the average, suggesting developmental advancement.

Categorical variables were summarized using frequency counts, relative frequencies, and valid percentages. Continuous variables were presented as means and SDs, along with minimum and maximum values, medians, and interquartile ranges (IQRs). Statistical analyses were performed using analysis of variance (ANOVA) through the general linear model (GLM), Spearman correlation test. For repeated measures, a GLM for repeated measures was used to model the impact of epilepsy surgery on clinical variables, which were considered as independent factors in the analysis.

A significance level of .05 or less was set as the threshold for statistical significance. All statistical analyses were conducted using IBM SPSS 23.0 for Windows (Statistical Package for the Social Sciences, 2021).

## 3 RESULTS

### 3.1 Presurgical evaluation

#### 3.1.1 Clinical variables

One‐hundred thirty patients were included in the study. There were 57 females (43.8%) and 73 males (56.2%). The median age at presurgical evaluation was 67 months (IQR = 67). The median age at seizure onset was 7.5 months (IQR = 18), and the median epilepsy duration was 49 months (IQR = 62). Most patients had severe DRE, with a median seizure frequency of 75 seizures per month (IQR = 270).

MRI revealed focal cortical dysplasia in 43 cases (33.1%), gliosis in 23 cases (17.7%), low‐grade tumors in 16 cases (12.3%), Rasmussen encephalitis in 15 cases (11.5%), malformations of cortical development in 14 cases (10.8%), tuberous sclerosis complex in six cases (4.6%), hippocampal atrophy in five cases (3.8%), and Sturge–Weber syndrome in three cases (2.3%). In five patients (3.8%), the MRI was negative.

#### 3.1.2 VABS before surgery

The average AE before surgery was 18.2 months (median = 17.1), indicating a very low level of adaptive functioning compared to their chronological age (CA), which averaged 78 months at the time of assessment. These scores reflect the age level at which an average individual in the normative sample would perform the same adaptive behaviors. For example, a communication AE score of 16.5 months suggests that the child's communication skills are like those of a 16.5‐month‐old child. The high discrepancy between CA and AE highlights the severe deficits in adaptive behavior among these children. The average AE scores were 16.5 months for communication, 18.1 months for socialization, and 20.2 months for daily living skills. For motor skills, the average AE score, based on 67 patients, was 16.4 months.

To assess the degree of developmental delay, the disparity between mean CA and AE was calculated. On average, there was a notable 57.3‐month gap between AE and CA. Specifically, the mean CA was 75.5 months (6 years 3 months), whereas the mean AE was 18 months (1 year 6 months). The median *z*‐score was −2.46 (range = −6 to −1.5), indicating that the AE was at least 1.5 SD below the average chronological age for all participants in the study.

**FIGURE 1:**
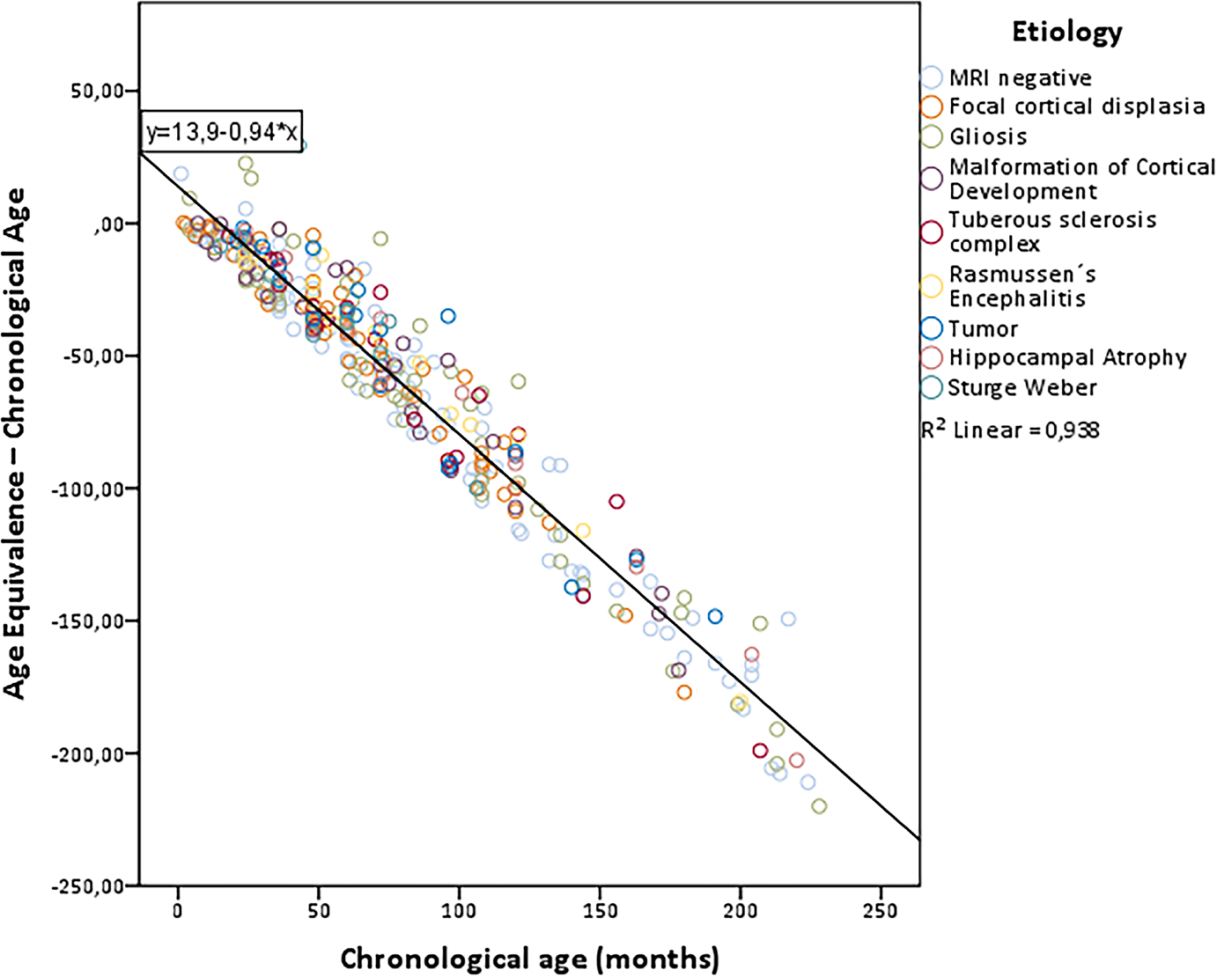
Correlation between gap in chronological age and age equivalence, and chronological age (Spearman rho = −.968, *p* < .001). MRI, magnetic resonance imaging.

Figure 1 shows that the gap between AE and CA widens as chronological age increases. Statistical analysis revealed a significant negative correlation between chronological age and the standardized *z*‐scores of adaptive behaviors, with a correlation coefficient of −.968 (*p* < .001). This strong negative correlation indicates that as chronological age increases, *z*‐scores decrease, suggesting greater developmental delays in older children relative to their peers.

Analysis of the degree of delay among the 130 patients revealed severe or profound impairment in all cases. Specifically, 55 patients (42.3%) were classified as having severe delay, and 75 patients (57.7%) exhibited profound delay. Although moderate delays were expected, none were encountered in our cohort. These findings confirm a high incidence of significant adaptative developmental delay in this cohort since the presurgical evaluation.

#### 3.1.3 AE between etiological groups

An ANOVA was conducted to examine potential differences in AE obtained with the VABS across different etiological groups. The results indicated significant differences between the etiology groups (one‐way ANOVA, *F* = 4.425, *p* < .001). Post hoc analysis using the Games–Howell test revealed that AE in both the hippocampal atrophy group and the Rasmussen encephalitis group is significantly higher (*p* = .034) compared to the gliosis group (*p* = .007). However, when controlling for age at evaluation, no significant differences were found among the etiological groups regarding the gap between CA and AE (one‐way ANOVA, *F* = 1.543, *p* = .227).

#### 3.1.4 VABS evaluations after surgery

Of the 130 patients submitted to surgery, 121 (93%) had a second VABS assessment after surgery. The average age at the time of assessment was 96 months (median = 84). The median time elapsed between the presurgical evaluation and the first postsurgical evaluation was 15 months (1 year 3 months).

During the first postsurgical evaluation, the average AE score was significantly higher, totaling 24.1 months (median = 20), compared to 18.2 months (median = 17.1) before surgery (*p* < .01, Wilcoxon test for dependent samples). The average AE scores were 22.0 months for communication, 24.6 months for socialization, and 26.0 months for daily living skills. For motor skills, the average AE score, based on 67 patients, was 20.6 months.

On average, children showed a 6‐month increase in AE on the first VABS assessment after surgery. However, although AE increased postsurgery, the gap between AE and CA continued to widen, as CA grew faster than AE. The average gap between AE and CA increased to 72.1 months (median = 59) postsurgery, compared to 58 months (median = 48.5) during the presurgical evaluation.

A second postoperative assessment using the VABS was undergone in 90 patients after epilepsy surgery. The average time elapsed between the presurgical evaluation and the first postsurgical evaluation was 34 months. The average CA of the evaluated patients was 116.4 months (9 years 7 months). During the second postsurgical evaluation, the average VABS score was also higher, totaling 27.5 months (median = 25.5). The results of the assessment indicated the following average scores for adaptive behavior domains: communication, 24.5; socialization, 29.2; daily skills, 30.5; and motor skills, 24.2.

Again, although the AE had increased from 18.2 preoperatively, to 24.1 at the first evaluation after surgery, to 27.5 months at the second evaluation after surgery, the gap between AE and CA widened to 88.7 months (median = 75), as the increase in CA outpaced the improvement in AE.

Analyzing the patients' scores individually, 18 participants showed a reduction in the gap between chronological age and AE in the first postoperative evaluation. However, only two patients continued to show this improvement in the second postoperative evaluation. This finding represents a small group with improvements and a positive impact on the assessed scores.

#### 3.1.5 Postsurgical seizure outcome

The resective surgical procedures performed were hemispherotomy in 68 cases (52.3%), lesionectomy in 27 cases (20.8%), lobectomy in 16 cases (12.3%), disconnection in 16 cases (12.3%), and multilobectomy in three cases (2.3%).

After surgery, 61 of 130 patients (46.9%) became seizure‐free (Engel class I), 30 patients (23.1%) had rare disabling seizures (Engel class II), 32 patients (24.6%) had seizure reduction (Engel class III), and seven patients (5.4%) had no reduction of seizure frequency (Engel class IV).

#### 3.1.6 Effect of seizure outcome on participants' adaptative score

We conducted a mixed between–within‐subjects ANOVA to assess the impact of seizure outcomes on participants' VABS scores across three time periods (presurgical, postsurgical I, and postsurgical II). The independent variables were time after surgery and seizure outcome (good seizure outcome vs. poor seizure outcome).

**FIGURE 2:**
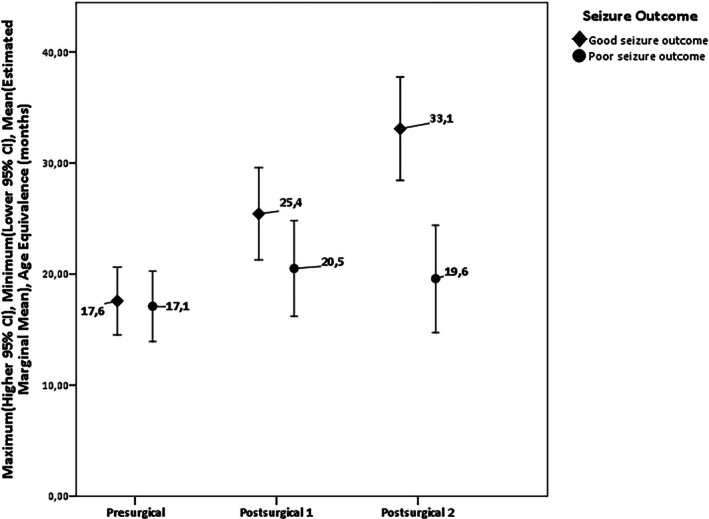
Analysis of variance to assess the impact of seizure outcomes on participants' Vineland Adaptive Behavior Scale scores across three time periods. CI, confidence interval.

The within‐subjects analysis showed a large effect for time after surgery, with patients showing an increase in AE scores across the three time periods (*F* = 26.03, *p <* .001, partial *η* ^2^ = .248). However, the between‐subjects analysis showed that the good seizure outcome group had a significant improvement when compared with the poor seizure outcome group (*F* = 5.845, *p =* .018, partial *η* ^2^ = .069). As seen in Figure 2, mean AE reached 33.1 months in the good seizure outcome group versus 19.6 months in the poor seizure outcome group.

We also considered the potential effect of ASM withdrawal on AE after surgery. Multivariate tests (Pillai's trace, Wilks' lambda, Hotelling's trace, and Roy's largest root) demonstrated a statistically significant main effect of surgery on AE across time points (*F*_2, 77_ = 20.87, *p* < .001, partial *η* ^2^ = .352), indicating a large effect size that accounted for 35.2% of the variance. Furthermore, a significant interaction emerged between surgery and seizure outcomes (*F*_2, 77_ = 10.15, *p* < .001, partial *η* ^2^ = .209), with good seizure outcome patients demonstrating greater AE compared to non‐seizure‐free patients.

In contrast, the analysis revealed no significant interaction between surgery and ASM withdrawal (*F*_2, 77_ = .16, *p* = .852, partial *η* ^2^ = .004), suggesting that the positive effects of surgery on AE were not influenced by medication discontinuation.

## 4 DISCUSSION

This retrospective study evaluated the long‐term adaptive outcomes of pediatric patients with DRE who underwent surgical treatment. The findings highlight the significant positive impact that epilepsy surgery has on adaptive behavior, especially in patients who achieved seizure freedom. Remarkably, the benefit was evident even in patients with severely impaired developmental baselines, where significant delays in adaptive functioning were present prior to surgery. Using the VABS as the primary assessment tool, we documented notable improvements in communication, socialization, daily living, and motor skills following surgery, even among those with severe preoperative impairments, showcasing the potential of surgical intervention in this highly vulnerable population.

Before surgery, most patients exhibited profound developmental delays, as demonstrated by the substantial gap between CA and AE scores. On average, the 57.3‐month gap between AE and CA underscores the detrimental effects of prolonged, uncontrolled epilepsy on neurodevelopment. This observation is consistent with existing literature, which highlights the strong association between early onset with extensive brain injuries,^26^, ^27^ severe cognitive and developmental impairments,^28^, ^29^ and DRE.^30^

### 4.1 Postoperative I

Our analysis revealed significant improvements in adaptive functioning following epilepsy surgery, particularly among patients who achieved seizure freedom (Engel class I). In the first postsurgical evaluation, conducted approximately 15 months after surgery, patients showed a 7.85‐month gain in AE scores across all VABS domains. The most notable improvements were seen in communication and daily living skills, domains typically most affected by DRE.

Our findings indicate that surgical treatment had a significant impact on adaptive outcomes, particularly in seizure‐free patients. Of the 130 patients who underwent resective surgery, the remarkable adaptive progress observed 15 months postsurgery was directly attributable to seizure freedom (Engel class I), independent of other clinical and demographic factors such as etiology, seizure frequency, epilepsy duration, and seizure onset.

Among our cohort, 61 patients (46.9%) achieved seizure freedom, whereas 69 (53.1%) continued to experience seizures (Engel classes II–IV). These findings are consistent with previous studies, which report seizure remission rates between 52% and 80.3%.^10^, ^11^, ^12^, ^13^, ^14^, ^15^ Based on these results, we conclude that effective seizure control, achieved by successful removal of the epileptogenic area, is closely linked to improved adaptive outcomes. However, it is important to acknowledge that the brain requires time to reorganize following surgery, and the extent of recovery depends on factors such as the nature of the lesion, location of surgery, and potential damage to functional areas during the procedure.

For patients who continued to experience seizures (Engel classes II–IV), it is likely that their extensive, diffuse, and early onset lesions impaired critical brain regions, making seizure control and functional reorganization more challenging.

### 4.2 Impact of seizure control on adaptive skills

Epilepsy surgery had a significant positive effect on adaptive skills during the initial postoperative evaluation, particularly in seizure‐free patients. The improvement in adaptive skills shortly after surgery is likely due to the reversal of epileptic encephalopathy, a phenomenon particularly relevant for patients with early onset epilepsy, which increases the risk of adaptive and behavioral alterations. In patients who did not achieve seizure control, improvements were modest, with a mean increase of only 3.4 months in AE, which was not statistically significant.

Several studies have confirmed that significant changes in adaptive skills and cognitive function occur within 12 months after epilepsy surgery in seizure‐free patients (Engel class I).^15^, ^17^, ^31^, ^32^, ^33^, ^34^, ^35^ However, some studies observed a temporary plateau in cognitive development during the initial postoperative evaluation, suggesting that further development may resume after a period of stabilization.^18^, ^36^, ^37^

The long‐term use of ASMs should also be considered. Following seizure control, a gradual reduction or withdrawal of ASMs can alleviate their cognitive side effects, potentially facilitating continued cognitive development.^37^ In contrast, patients who continued to experience seizures likely did not have their medication regimen adjusted, which could explain the lack of improvement in AE, as seen in previous studies.^38^, ^39^

### 4.3 Postoperative II

At the second postoperative evaluation, conducted 34 months after surgery, our study reaffirmed the sustained positive impact of epilepsy surgery on patients who remained seizure‐free. Improvements in cognitive profiles and adaptive skills, as assessed by the VABS, were evident in these patients. The study by Ueda et al. also indicated that seizure‐free patients experienced a greater increase in AE in specific adaptive skills, such as the communication domain (social interaction, safety, etc.), which were associated with improvements in autism spectrum disorder symptoms and executive function. This suggests that seizure control facilitates adaptive gains. In other words, both studies highlight that seizure control is a key factor in improving adaptive functioning. This underscores the importance of effective resective surgery in enhancing not only epilepsy control but also daily life skills.^40^

Although our study did not specifically assess cognitive processing speed, we noted that seizure‐free patients exhibited sustained gains in AE, even after long‐term epilepsy. In contrast, other studies report that developmental scores remain stable during the postoperative period, reflecting a plateau in cognitive trajectories.^36^, ^41^

### 4.4 Patients with ongoing seizures

Patients who continued to experience seizures showed minimal improvements in adaptive skills even after 4 years of follow‐up. The gain in AE for this group was only approximately 3 months, and a significant portion of these patients (53.1%) failed to achieve seizure control (Engel class II–IV). Additionally, many patients already exhibited severe impairments before surgery, with approximately 31.5% of the cohort showing severely compromised VABS scores.

Our results indicate that ongoing seizures exacerbate these conditions, with 31.6% of patients demonstrating severe to profound adaptive delays during the second postoperative evaluation. These findings align with the literature, suggesting that adaptive improvements are unlikely for patients with ongoing seizures, particularly those with severe preoperative impairments.^10^, ^42^, ^43^

Several factors likely contributed to the poor prognosis observed in patients who did not achieve seizure freedom. First, epileptic encephalopathy may not have been fully reversed, hindering improvements in adaptive functions. Additionally, factors such as early onset seizures, high seizure frequency, and extensive brain damage could have impeded normal development by disrupting neuroplasticity mechanisms.

Another factor is the period of brain reorganization that occurs after surgery. The recovery process is influenced by residual function in the epileptogenic zone, the impact of epilepsy on the opposite hemisphere, and postsurgical complications. It is possible that patients who did not achieve seizure freedom had a higher proportion of congenital pathologies compared to acquired conditions.

### 4.5 Limitations of the study

Although the study yielded promising results, several limitations should be acknowledged. The retrospective nature of the study introduces potential selection biases, as patients underwent surgery over a 23‐year period, during which surgical techniques and postoperative care may have evolved. Additionally, the reduced sample size for the second postoperative evaluation due to loss to follow‐up may limit the generalizability of our findings.

The heterogeneity of the patient population, with various etiologies, seizure types, and surgical procedures, is another limitation. Although multivariate analysis was used to control for some variables, specific factors unique to certain patient groups may have influenced the results. Future studies with larger, more homogeneous cohorts are needed to confirm our findings.

Regarding loss to follow‐up, patients from all Brazilian states are referred to our institution for epilepsy surgery. As a result, geographical and economic barriers prevented some patients from returning for follow‐up visits beyond 2 years postoperatively. In our protocol, we made up to three attempts to schedule evaluations, consultations, and examinations for each patient. Additionally, among the initial 130 children, 121 returned for the first postoperative VABS evaluation and 90 for the second evaluation. During the follow‐up period, only three of 130 patients died (two due to pneumonia‐related complications and one due to sudden unexpected death in epilepsy). This information underscores that loss to follow‐up was primarily attributable to logistical challenges rather than adverse outcomes.

As for the VABS, we used the VABS II through a semistructured interview conducted individually with parents or caregivers. Recently, the VABS III (new version) was translated from English to Portuguese. This translation is available for use in Brazil; however, the publication did not include studies with a Brazilian sample through validation and cross‐cultural adaptation. We recognize the need for future studies to ensure greater accuracy and clinical applicability.

Another limitation of our study is the absence of a control group. However, it is important to note that our study was specifically designed to compare outcomes within a surgical cohort, namely, patients with seizure control (Engel class I) versus those without seizure control (Engel classes II–IV). This intracohort comparison allowed us to investigate how seizure control relates to changes in neuropsychological outcomes (e.g., VABS scores) following surgery. Our intent was not to compare surgical outcomes with those of a purely medically managed group, but rather to explore differential outcomes among patients who underwent surgical intervention.

We recognize that including a third nonsurgical control group could provide additional insights into the natural history of adaptive functioning in pediatric patients with DRE. However, ethical and practical challenges—such as the difficulty in creating a well‐matched control group when surgery is the standard of care—limit the feasibility of such an approach, especially considering that we would need to bring in the patients from the control group every 2 years to perform a VABS evaluation.

### 4.6 Clinical and practical implications

The findings underscore the potential benefits of surgical intervention in children with DRE. Given the association between epilepsy duration and cognitive outcomes, early identification and surgical referral are crucial to prevent further cognitive decline.

Seizure freedom is a key factor in maximizing adaptive outcomes postsurgery. Comprehensive preoperative evaluation and precise localization of the epileptogenic zone are essential for achieving seizure freedom and optimizing adaptive recovery.

## 5 CONCLUSIONS

Our study demonstrates that epilepsy surgery offers significant benefits in terms of adaptive functioning for pediatric patients with DRE, even among those with severe neurological impairments. Despite the profound developmental challenges many patients faced, surgery proved beneficial, especially for those who achieved seizure freedom. However, although adaptive improvements were observed, the widening gap between CA and AE highlights the importance of early intervention to maximize developmental outcomes. These findings support the growing body of evidence advocating for surgical treatment in cases of refractory epilepsy, highlighting the potential for positive outcomes even in patients with severe preoperative deficits. Further research is needed to refine treatment strategies, optimize patient outcomes, and improve long‐term cognitive recovery in this vulnerable population.

## AUTHOR CONTRIBUTIONS

**Ana Valeria Duarte Oliveira:** Conceptualization (lead); formal analysis (equal); funding acquisition (supporting); investigation (lead); methodology (supporting); writing—original draft (lead); writing—review and editing (lead). **Ana Paula Hamad:** Project administration (lead); writing—review and editing (supporting). **Antonio Carlos dos Santos:** Validation (supporting); visualization (equal); writing—original draft (supporting). **Américo Ceiki Sakamoto:** Software (lead); writing—review and editing (supporting). **Geisa de Angelis:** Investigation (supporting); writing—original draft (supporting). **Hélio Rubens Machado:** Conceptualization (supporting); formal analysis (equal); writing—review and editing (supporting). **João Pereira Leite:** Writing—original draft (supporting); writing—review and editing (supporting). **Lauro Wichert‐Ana:** Visualization (equal); writing—review and editing (supporting). **Marcelo Volpon Santos:** Visualization (equal); writing—original draft (supporting); writing—review and editing (supporting). **Tonicarlo Rodrigues Velasco:** Conceptualization (supporting); data curation (lead); formal analysis (equal); funding acquisition (lead); methodology (lead); supervision (lead); writing—original draft (supporting); writing—review and editing (supporting). **Úrsula Thomé:** Resources (equal); validation (lead); writing—review and editing (supporting).

## FUNDING INFORMATION

This study was funded by Coordination of Improvement of Higher Education Personnel (Capes) ‐ Brazil (Centro de Aperfeiçoamento em Pesquisa e Ensino; financing code 001; grant number 88882.328290/2019‐01).

## CONFLICT OF INTEREST STATEMENT

None of the authors has any conflict of interest to disclose.

## ETHICS STATEMENT

We confirm that we have read the Journal's position on issues related to ethical publication and affirm that this report is consistent with those guidelines. All procedures performed in studies involving human participants were in accordance with the ethical standards of the institutional and/or national research committee and with the 1964 Helsinki declaration and its later amendments or comparable ethical standards.

## PATIENT CONSENT STATEMENT

Informed consent was obtained from all individual participants included in the study.

## PERMISSION TO REPRODUCE MATERIAL

The material contained in this article may be reproduced in other sources, provided that proper citation is given, and copyright is respected.

## CLINICAL TRIAL REGISTRATION

This study was submitted to research ethics committee of the Clinical Hospital of the Medical School of Ribeirão Preto–University of São Paulo and received the approved opinion number 4.829.087.

## Data Availability

The data related to this article will be available for public access through a link to the chosen institutional repository.
